# Deletion of the WD40 domain of ATG16L1 exacerbates acute pancreatitis, abolishes LAP-like non-canonical autophagy and slows trypsin degradation

**DOI:** 10.1080/15548627.2024.2392478

**Published:** 2024-08-31

**Authors:** Michael Chvanov, Svetlana Voronina, Matthew Jefferson, Ulrike Mayer, Robert Sutton, David N. Criddle, Thomas Wileman, Alexei V. Tepikin

**Affiliations:** a Department of Molecular & Clinical Cancer Medicine, Institute of Systems Molecular & Integrative Biology, University of Liverpool, Liverpool, UK; b Biomedical Research Centre, School of Biological Sciences, University of East Anglia, Norwich, UK; c Liverpool University Hospitals NHS Foundation Trust, Liverpool, UK; d Norwich Medical School, University of East Anglia, Norwich, UK

**Keywords:** Amylase, caerulein, cholecystokinin, endocytic vacuoles, LC3-associated phagocytosis, palmitoleic acid

## Abstract

The WD40 domain (WDD) of ATG16L1 plays a pivotal role in non-canonical autophagy. This study examined the role of recently identified LAP-like non-canonical autophagy (LNCA) in acute pancreatitis. LNCA involves rapid single-membrane LC3 conjugation to endocytic vacuoles in pancreatic acinar cells. The rationale for this study was the previously observed presence of trypsin in the organelles undergoing LNCA; aberrant trypsin formation is an important factor in pancreatitis development. Here we report that the deletion of WDD (attained in ATG16L1[E230] mice) eliminated LNCA, aggravated caerulein-induced acute pancreatitis and suppressed the fast trypsin degradation observed in both a rapid caerulein-induced disease model and in caerulein-treated isolated pancreatic acinar cells. These experiments indicate that LNCA is a WDD-dependent mechanism and suggest that it plays not an activating but a protective role in acute pancreatitis. Furthermore, palmitoleic acid, another inducer of experimental acute pancreatitis, strongly inhibited LNCA, suggesting a novel mechanism of pancreatic lipotoxicity.

**Abbreviation:** AMY: amylase; AP: acute pancreatitis; CASM: conjugation of Atg8 to single membranes; CCK: cholecystokinin; FAEE model: fatty acid and ethanol model; IL6: interleukin 6; LA: linoleic acid; LAP: LC3-associated phagocytosis; LMPO: lung myeloperoxidase; LNCA: LAP-like non-canonical autophagy; MAP1LC3/LC3: microtubule-associated protein 1 light chain 3; MPO: myeloperoxidase; PMPO: pancreatic myeloperoxidase; POA: palmitoleic acid; WDD: WD40 domain; WT: wild type.

## Introduction

Acute pancreatitis (AP) is a frequent disease associated with considerable morbidity and significant mortality [1]. The disease involves malfunctioning of several pancreatic components and cell types (e.g [2,3]). Notably, damage of pancreatic acinar cells is considered as the initiating event of AP (reviewed in [4,5]). The primary mechanism of such damage is currently debated and may include intracellular trypsinogen activation with subsequent autodigestion of the organ [6–10], Ca^2+^ overload [11–13], mitochondrial damage [14,15], NFKB/NF-kB activation [16–18], disruption of macroautophagy/autophagy [19,20], aberrant exocytosis and endocytosis [21–24] or a combination of these factors (reviewed in [4,5,25,26]). Autodigestion of the pancreas and intrapancreatic trypsin formation are of particular relevance to this investigation. Under normal physiological conditions the trypsin precursor trypsinogen is secreted by the exocrine pancreas and activated in the upper part of the small intestine. Aberrant intrapancreatic trypsinogen activation (i.e. trypsin formation) is considered a hallmark of the disease, although the specific role of intrapancreatic trypsin in the initiation of AP is debated extensively (see [7,8,10,27]). Recent studies from the Sahin-Toth laboratory have demonstrated convincingly that aberrant intracellular trypsin formation is sufficient to trigger AP [6,7,28]. In animal models of AP intracellular trypsinogen activation is amongst the earliest manifestations of pancreatic tissue damage (reviewed in [29]). Another early indication of the cell damage in AP is formation of intracellular vacuoles, with several types described in damaged acinar cells [19,21,23,30,31]. The endocytic vacuoles are prominent contributors to vacuolization. These organelles rapidly form as a result of aberrant compound exocytosis triggered by the Ca^2+^-releasing inducers of AP [23,24,32] which involves fusion of multiple secretory granules followed by retrieval of post-endocytic structures [23,24]. The vacuoles can reach 10 µm in diameter; this requires anomalous fusion of hundreds of secretory granules [24]. Digestive enzymes and precursors of digestive enzymes are partially retained in the endocytic vacuoles [23,24,33]. Formation of trypsin (i.e. activation of trypsinogen) has been observed in the endocytic vacuoles [23,33]. Furthermore, the vacuoles can rupture, spilling trypsin into the cytosol and precipitating cell death [24]. Dysregulation of autophagy is considered to be as a significant factor in the initiation and progression of AP (e.g [19,21,34]. reviewed in [35,36]). Recent studies have described a degradative pathway related to autophagy termed LC3-associated phagocytosis (LAP) ([37,38] reviewed in [39]). LAP involves direct LC3 conjugation to the organellar membrane (without prior formation of a phagophore). A similar mechanism termed CASM (conjugation of Atg8 to single membranes) was described by Florey and colleagues [40–42]. The current study is based on the recent finding by our group that endocytic vacuoles (trypsinogen-activating organelles) undergo fast LAP-like non-canonical autophagy (LNCA), which shares some (e.g., fast single-membrane LC3 conjugation) but not all properties of LAP and CASM [33,36].

The aim of the study was to evaluate the potential role of LNCA in AP. To elucidate the putative role of LNCA we utilized ATG16L1[E230] mice; ATG16L1 is required for both canonical and non-canonical autophagy, however different domains of this protein are utilized in these different types of autophagy. In ATG16L1[E230] mice the WDD of ATG16L1 required for LAP and LNCA is missing, although the N-terminal amino acids required for canonical autophagy are retained [43,44]. *In vivo* AP models (caerulein model of AP and the FAEE model of AP combining palmitoleic acid [POA] and ethanol) were applied to LNCA-deficient mice and their wild-type (WT) littermates to define the role of LNCA in the early stages of AP. These models were accompanied by *in vitro* studies of LC3 conjugation to endocytic vacuoles and cell death in isolated pancreatic acinar cells.

## Results

### LC3 conjugation to endocytic vacuoles is suppressed in WDD-deficient ATG16L1[E230] mice

**Figure 1.**
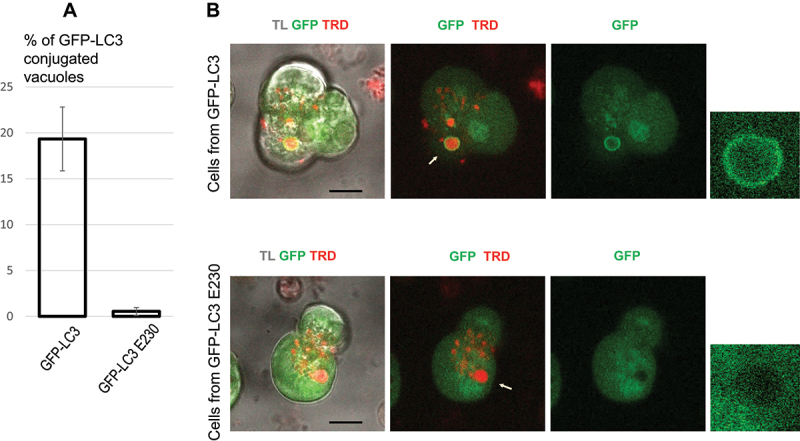
GFP-LC3 conjugation to endocytic vacuoles in pancreatic acinar cells isolated from GFP-LC3 mice and GFP-LC3 ATG16L1[E230] mice. Cells were incubated in physiological HEPES-buffered solution containing 100 pM CCK and Texas Red dextran (TRD) for 30 min at 35°C. GFP-LC3 ATG16L1[E230] is abbreviated to GFP-LC3 E230 on the graph. In this experiment we analyzed 425 vacuoles from 138 GFP-LC3 cells (79 GFP-LC3-conjugated vacuoles observed) and 596 vacuoles from 177 GFP-LC3 ATG16L1[E230] cells (only 2 GFP-LC3-conjugated vacuoles observed). In these experiments 5 GFP-LC3 mice and 6 GFP-LC3 ATG16L1[E230] mice were utilized for cell isolation. (A) the bars reveal the proportion of GFP-LC3-conjugated vacuoles (mean values ± standard error). (B) shows an example of a GFP-LC3-conjugated endocytic vacuole in an acinar cell isolated from a GFP-LC3 mouse and the lack of GFP-LC3 conjugation to an endocytic vacuole in an acinar cell isolated from a GFP-LC3 ATG16L1[E230] mouse. GFP fluorescence is shown in green color. Endocytic vacuoles were revealed by the fluorescence of endocytosed TRD (red color). TL on the left panels indicates transmitted light. Scale bars: 10 µm. Expanded fragments, containing vacuoles indicated by white arrows, are shown on the right panels.

High “pathophysiological” concentrations of the Ca^2+^-releasing hormone cholecystokinin (CCK) induce formation of large endocytic vacuoles in pancreatic acinar cells [23,45], followed by LC3 conjugation to these vacuoles [33]. In our previous study [33] we observed strong inhibition of LC3 conjugation to endocytic vacuoles in the acinar cells from ATG16L1[E230] mice. In these previous experiments we utilized replication deficient adenovirus to express mCherry-LC3 in the acinar cells. In the current study we confirmed these results by generating GFP-LC3 ATG16L1[E230] mice and comparing LC3 conjugation in these animals with that in GFP-LC3 mice. We observed that in pancreatic acinar cells from the mutant GFP-LC3 ATG16L1[E230] mice GFP-LC3 recruitment to endocytic vacuoles, formed in response to 100 pM CCK, was almost completely inhibited (see Figure 1). Almost complete inhibition of GFP-LC3 conjugation was also observed in similar experiments with 10 nM CCK where analysis of 708 vacuoles from 163 GFP-LC3 cells revealed 69 GFP-LC3- conjugated vacuoles, while conjugation occurred only once in 704 vacuoles from 149 GFP-LC3 ATG16L1[E230] cells (not shown). These experiments confirm that LC3 is recruited to endocytic vacuoles in pancreatic acinar cells by LNCA rather than canonical autophagy, and that this recruitment is almost completely blocked in the GFP-LC3 ATG16L1[E230] mice. We next utilized ATG16L1[E230] mice in animal models of AP.

### Caerulein-induced acute pancreatitis is more severe in WDD and LNCA-deficient ATG16L1[E230] mice

**Figure 2.**
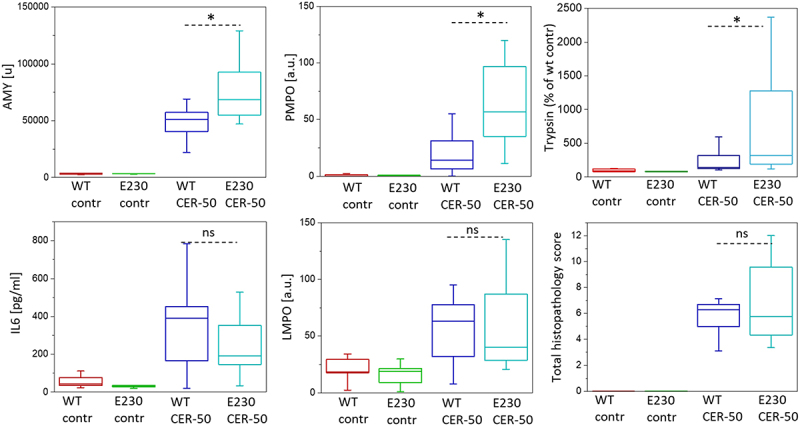
Severity of acute pancreatitis induced by a high dose of caerulein in wild-type and ATG16L1[E230] mice. The figure shows parameters characterizing the severity of acute pancreatitis (AP) in mice with deficient non-canonical autophagy (ATG16L1[E230] mice, abbreviated to E230 on the graph) and wild-type littermates (WT). Experimental AP was induced by 7 hourly intraperitoneal injections of caerulein (50 µg/kg); 12 ATG16L1[E230] mice and 13 WT mice were utilized in these experiments. Animals were humanely sacrificed 8 h after the first injection. Control experiments involved intraperitoneal injections of vehicle solution without caerulein (5 ATG16L1[E230] mice and 5 WT mice were used). Symbols above the bars illustrate the outcome of a Mann-Whitney test; symbol * indicates statistical significance (p < 0.05), ns indicates that the difference was not statistically significant. Specific *p* values were the following: serum AMY p = 0.011, pancreatic MPO (PMPO) p = 0.002, pancreatic trypsin p = 0.041, IL6 p = 0.37, lung MPO (LMPO) p = 0.64, total histopathology score p = 0.55 (information about the components of the histopathology score is summarized in Figure S2).

**Figure 3.**
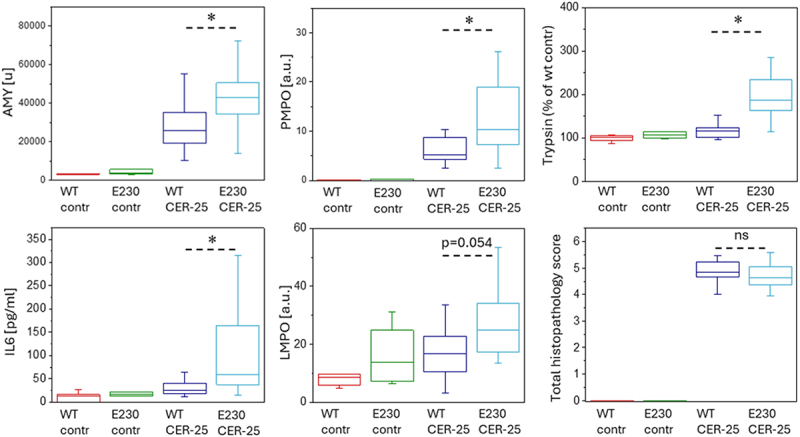
Severity of acute pancreatitis induced by a moderate dose of caerulein in wild-type and ATG16L1[E230] mice. The figure shows parameters characterizing the severity of acute pancreatitis (AP) in mice with deficient non-canonical autophagy (ATG16L1[E230] mice, abbreviated to E230 on the graph) and wild-type littermates (WT). Experimental AP was induced by 7 hourly intraperitoneal injections of caerulein (25 µg/kg); 12 ATG16L1[E230] mice and 18 WT mice were utilized in these experiments. Animals were humanely sacrificed 8 h after the first injection. Control experiments involved intraperitoneal injections of vehicle solution without caerulein (6 ATG16L1[E230] mice and 6 WT mice were used). Symbols above the bars illustrate the outcome of a Mann-Whitney test; symbol * indicates statistical significance (p < 0.05), ns indicates that the difference was not statistically significant. Specific *p* values were the following: serum AMY p = 0.047, pancreatic MPO (PMPO) p = 0.044, pancreatic trypsin p = 0.0002, IL6 p = 0.01, lung MPO (LMPO) p = 0.054, total histopathology score p = 0.65 (information about the components of the histopathology score is summarized in Figure S2).

Our in vivo experiments comparing the severity of AP between WT and ATG16L1[E230] mice employed the standard and most frequently used experimental model of AP induced by intraperitoneal injections of caerulein. Caerulein is an agonist of CCK receptors extensively utilized to cause hyperstimulation of the pancreas [29,46]. In the conventional high dose (50 µg/kg) caerulein model we observed substantial, statistically significant differences in important biochemical parameters characterizing the severity of AP; serum AMY (amylase), pancreatic MPO (myeloperoxidase; PMPO) and pancreatic trypsin in ATG16L1[E230] mice were increased in comparison with WT littermates (Figure 2). IL6 (interleukin 6) and lung MPO (LMPO) (biochemical parameters relevant to systemic damage) were not different (Figure 2). Total histopathology scores were also similar in WT and ATG16L1[E230] mice (Figure 2). While not all parameters characterizing the severity of AP reflected the difference between ATG16L1[E230] and WT mice, the significant increase of serum AMY (parameter utilized for the clinical diagnosis of AP) and trypsin (protease responsible for the autodigestion of the pancreas) in ATG16L1[E230] mice suggested a protective effect of LNCA. We hypothesized that the protective effect of LNCA would be particularly prominent in less severe forms of caerulein-induced AP and conducted the relevant *in vivo* models of this disease. Indeed, in the moderate model of AP (induced by caerulein injections of 25 µg/kg) the levels of serum AMY, intrapancreatic trypsin, PMPO and IL6 were substantially and significantly increased in WDD and LNCA-deficient ATG16L1[E230] mice (Figure 3), while the *p* value for LMPO was 0.054 (i.e. close to the threshold for statistical significance). Total histopathology scores were similar in WT and ATG16L1[E230] mice (Figure 3). In this moderate model of AP 4 out of 5 biochemical parameters commonly used for characterizing the severity of AP were significantly increased in WDD and LNCA-deficient ATG16L1[E230] mice compared to WT and one parameter (LMPO) was close to significant difference. Notably, PMPO (a parameter characterizing pancreatic inflammation) levels were consistently and significantly increased in LNCA-deficient ATG16L1[E230] mice (in both severe and moderate caerulein models, see Figures 2 and 3) further highlighting a protective role of LNCA in AP. Importantly, the moderate caerulein model (25 µg/kg) revealed the difference in trypsin levels between WT and ATG16L1[E230] mice. A difference in the pancreatic trypsin level was also observed in the mild caerulein model (induced by caerulein injections of 10 µg/kg, see Figure S1). Three other biochemical parameters characterizing the severity of AP (PMPO, LMPO and IL6) were statistically higher for WDD and LNCA-deficient ATG16L1[E230] mice compared to WT while the *p* value for AMY measurements was 0.08. Total histopathology scores were not significantly different between WT and WDD and LNCA-deficient ATG16L1[E230] mice, although, in the moderate and mild models the score for necrosis was higher for WDD and LNCA-deficient ATG16L1[E230] mice (Figure S2), consistent with a protective role for LNCA.

### Fast trypsin degradation in rapid caerulein AP model is suppressed in WDD and LNCA-deficient ATG16L1[E230] mice

**Figure 4.**
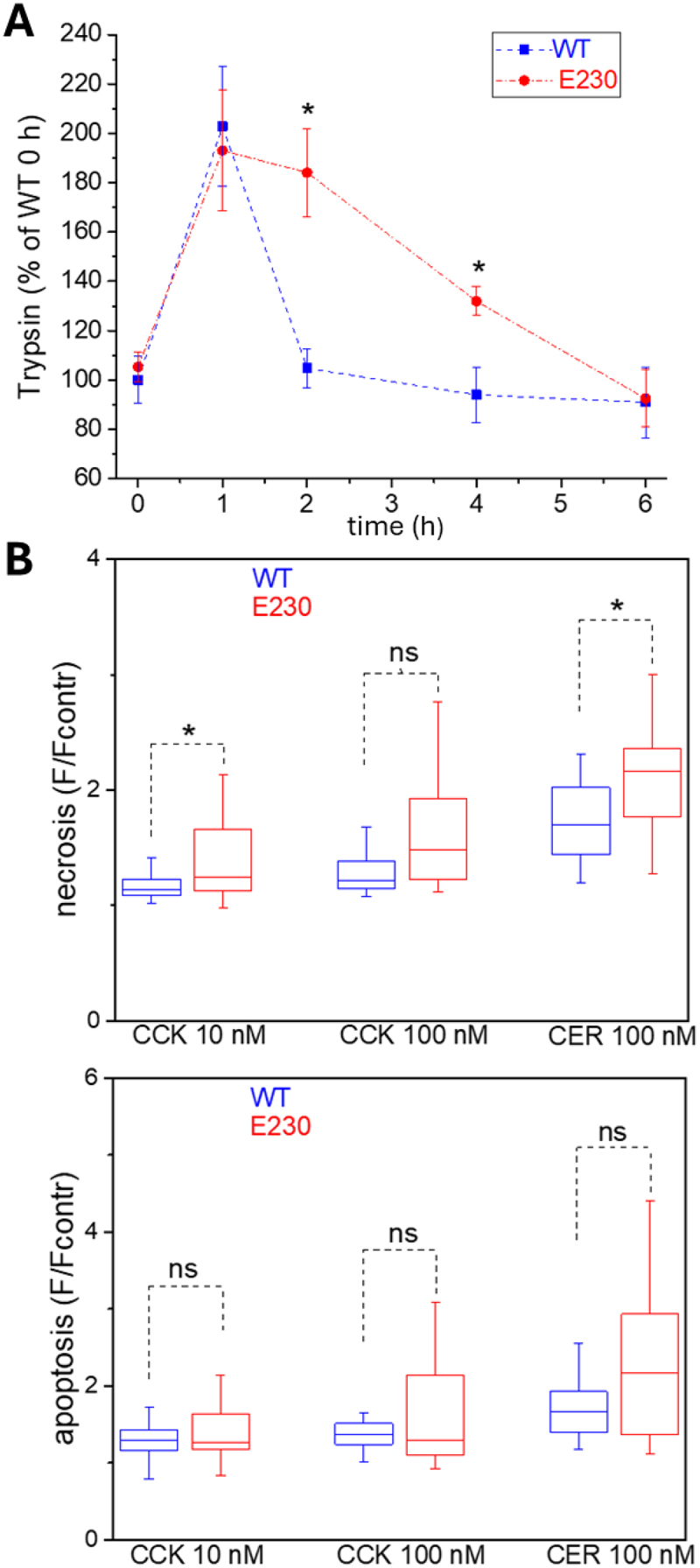
Deletion of WDD of ATG16L1 slows trypsin degradation and potentiates cell death. (A) rapid changes in the pancreatic trypsin levels in wild-type (WT) and ATG16L1[E230] (abbreviated to E230 on the graph) mice were induced by one intraperitoneal caerulein injection (50 µg/kg). Experiments were conducted on mice fed *ad libitum*. Trypsin levels were measured in pancreata obtained from untreated mice (i.e. before the caerulein injections, 0 h) as well as at 1 h, 2 h, 4 h and 6 h after the injection. Each time point on the graph shows the results of measurements (± standard error) obtained from 6 mice (normalized to the mean values from WT at 0 h for each of experiments). Symbol * indicates statistical significance (p < 0.05), ns indicates that the difference was not statistically significant. Trypsin levels at the 1 h time points were not significantly different between WT and E230 mice (p = 0.89). Note the substantial and statistically significant decline in the trypsin levels between 1 h and 2 h in WT mice (p = 0.005), and the absence of such decline in the WDD and LNCA-deficient E230 mice (p = 0.77). The significant difference in the trypsin levels between WT and ATG16L1[E230] developed at 2 h and disappeared at 6 h after the single injection. We have repeated critical early points of this experiment (0, 1 and 2 h) using mice fasted for 12 h (see Figure S3A). The rational for these additional experiments was to test putative interference from canonical autophagy that could be initiated in the fasted mice. Similar results were observed in experiments conducted on mice fed *at libitum* and fasted mice (Figure S3A). (B) apoptosis and necrosis of pancreatic acinar cells isolated from wild-type and ATG16L1[E230] mice. Apoptosis (right panel) and necrosis (left panel) were assessed at 14 h end point as the fluorescence of caspase 3/7 and propidium iodide, respectively. In these experiments 18 wild-type (WT) mice and 13 ATG16L1[E230] mice (E230) were utilized. The box plots (median line, box as first to third quartile, and whiskers as 1.5 times inter-quartile range) show the fluorescence measurements of the treatment groups divided by the fluorescence recorded in the corresponding vehicle control group. The *p* values for apoptosis were the following: 0.79 for CCK 10 nM, 0.98 for CCK 100 nM and 0.09 for cerulein (CER) 100 nM. The *p* values for necrosis were the following: 0.043 for CCK 10 nM, 0.054 for CCK 100 nM and 0.040 for cerulein (CER) 100 nM.

An increased pancreatic trypsin level in WDD and LNCA-deficient ATG16L1[E230] mice was the consistent feature of all 3 *in vivo* models of caerulein-induced AP (Figures 2, 3 and S1). Both intrapancreatic trypsinogen activation and LNCA are fast processes ([47,48] and [33] correspondingly). It was therefore important to test the relationships between these responses at the early stages of experimental AP. We conducted a rapid *in vivo* model of AP utilizing one intraperitoneal injection of caerulein (50 µg/kg) and measured pancreatic trypsin levels at 1, 2, 4 and 6 h after injection. We found that at 1 h, there was no resolvable difference between the trypsin levels in WT and ATG16L1[E230] mice. However, a strong, statistically significant difference developed at 2 h (Figures 4A and S3A), highlighting the protective role of LNCA and its contribution to the recovery phase of the first rapid peak of trypsin formation in the caerulein model of AP. The significant difference between the WT and ATG16L1[E230] mice was still observed at 4 h post-injection but disappeared at 6 h, suggesting that by that time other slower processes of trypsin removal were active. The animal models, described in this section, were paralleled by experiments on isolated pancreatic acinar cells (Figure S3B). Similarly, to the animal model, there was no resolvable difference at 1 h, while significant difference developed at 2 h. In the isolated cells the difference disappeared at 4 h after caerulein addition (i.e. somewhat earlier than in the animal model).

### The role of WDD and LNCA in CCK/caerulein-induced cell death of pancreatic acinar cells

At the cellular level, the damaging effects of the AP inducers and protective or aggravating effects of genetic modifications can be evaluated by the degree of acinar cell necrosis and apoptosis. We used pancreatic acinar cells isolated from WDD and LNCA-deficient ATG16L1[E230] and WT mice to probe the putative role of LNCA in the cell death triggered by CCK and caerulein, applying high doses of these Ca^2+^-releasing agonists (Figure 4B). In this conventional *in vitro* model of AP, statistically significant increases in necrosis induced by 10 nM CCK (*p* = 0.043) and 100 nM caerulein (*p* = 0.040) were observed in the cells isolated from WDD and LNCA-deficient ATG16L1[E230] mice (Figure 4B). The median value of necrosis measurements for ATG16L1[E230] cells treated with CCK 100 nM was also higher than for WT cells, although statistical significance was not attained (*p* = 0.054, Figure 4B), while there was no resolvable difference in apoptosis of cells treated with CCK or caerulein (Figure 4B). The observed difference in the CCK- and caerulein-induced necrosis is consistent with the protective effect of LNCA.

### Palmitoleic acid inhibits LAP-like non-canonical autophagy in pancreatic acinar cells

**Figure 5.**
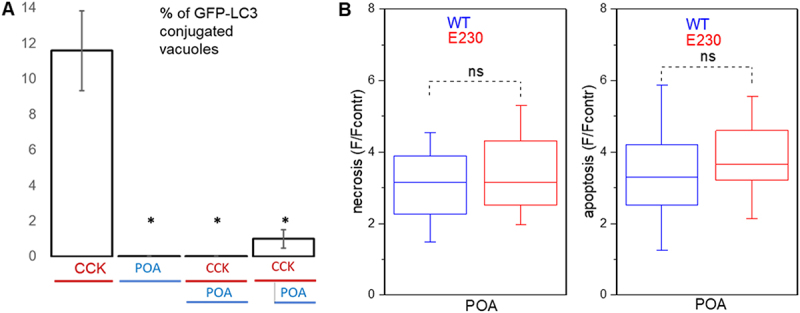
Effect of palmitoleic acid on LAP-like non-canonical autophagy and cell death of pancreatic acinar cells isolated from wild-type and ATG16L1[E230] mice. (A) effect of palmitoleic acid on GFP-LC3 conjugation to endocytic vacuoles in pancreatic acinar cells. Cells were isolated from GFP-LC3 transgenic mice and incubated in physiological HEPES-buffered solution containing Texas red dextran (TRD) at 35°C. Symbol * indicates statistically significant difference from the values obtained in experiments with 10 nM CCK (without POA). The bars reveal the proportion of GFP-LC3-conjugated vacuoles (mean values ± standard error). Application of 10 nM CCK for 30 min resulted in the generation of endocytic vacuoles and GFP-LC3 conjugation to the endocytic vacuoles (405 vacuoles, 101 cells were analyzed, 5 mice were used for cell isolation in these experiments). In all experiments shown in this figure the extracellular solution contained 1% ethanol (utilized as a vehicle for POA). In separate experiments we found that this ethanol concentration had no effect on the number of vacuoles (p = 0.97, not shown) and the proportion of GFP-LC3-conjugated vacuoles (p = 0.92, not shown) produced by 10 nM of CCK; 665 vacuoles from 177 cells (derived from 9 mice) treated with 1% ethanol (added to the extracellular solution described in the section “isolation of pancreatic acinar cells”) and 708 vacuoles from 163 cells (derived from 6 mice) maintained in the ethanol-free extracellular solution, were utilized in these experiments. Notably, we did not observe GFP-LC3-conjugated endocytic vacuoles in cells treated with 200 µm palmitoleic acid (POA) (133 vacuoles, 61 cells were analyzed, 5 mice were used for cell isolation). Furthermore, addition of 200 µm POA to the solution containing 10 nM CCK completely inhibited GFP-LC3 conjugation to endocytic vacuoles (in this experiment both CCK and POA were applied simultaneously for 30 min (177 vacuoles, 71 cells were analyzed, 5 mice were used for cell isolation). We next conducted an experiment involving application of 10 nM CCK followed by the application of POA after a delay of 10 min (in the continuous presence of 10 nM CCK, POA was present for 20 min). In this configuration POA also strongly inhibited GFP-LC3 conjugation to the endocytic vacuoles (p = 0.008; 440 vacuoles; 130 cells were analyzed; 6 mice were used for cell isolation). Effects of lower concentrations of POA are shown on the figure S6. (B) effect of palmitoleic acid on apoptosis and necrosis of pancreatic acinar cells isolated from wild-type and ATG16L1[E230] mice. In these experiments we utilized 100 µm of POA. Apoptosis (right panel) and necrosis (left panel) were assessed at 14 h end point as the fluorescence of caspase 3/7 and propidium iodide, respectively. In these experiments 16 wild-type (WT) mice and 11 ATG16L1[E230] mice (E230) were utilized. The box plots (median line, box as first to third quartile, and whiskers as 1.5 times inter-quartile range) show the fluorescence measurements of the treatment groups divided by the fluorescence recorded in the corresponding vehicle control group; ns indicates that the difference was not statistically significant. The *p* value for apoptosis was 0.27. The *p* value for necrosis was 0.51.

Palmitoleic acid is a well-characterized inducer of acute pancreatitis in animal and cellular models (e.g [11,12,49]). We selected palmitoleic acid to test the putative role of WDD and LNCA in these models. The cellular study revealed a complete inhibition of LC3 conjugation to endocytic vacuoles induced by 200 µM of palmitoleic acid (i.e. complete inhibition of LNCA by palmitoleic acid, see Figure 5A). Treatment with palmitoleic acid resulted in a smaller number of endocytic vacuoles than treatment with CCK (Figure S4). Since LNCA was originally characterized in CCK-treated cells, we repeated experiments combining CCK and POA and observed complete inhibition of LC3 conjugation to endocytic vacuoles formed by the combined treatment (Figure 5A). Finally, we probed the effect of POA on the CCK-induced endocytic vacuoles formed prior to POA application. In these experiments we explored the brief time lag (approximately 15–20 minutes) between the formation and LC3 coating of CCK-induced vacuoles [33]. In this type of experiment POA almost completely inhibited LC3 conjugation to CCK-induced endocytic vacuoles (Figure 5A); similar results were obtained in experiments with linoleic acid (Figure S5).

### Deletion of WDD of ATG16L1 does not change the severity of fatty acid and ethanol models of acute pancreatitis

In the *in vitro* POA treatment model, there was no resolvable differences in apoptosis or necrosis between pancreatic acinar cells isolated from WT and from WDD and LNCA-deficient ATG16L1[E230] mice and treated by POA (Figure 5B). The absence of resolvable difference in this conventional cellular model could be explained by POA-induced LNCA inhibition in the acinar cells from WT mice.

**Figure 6.**
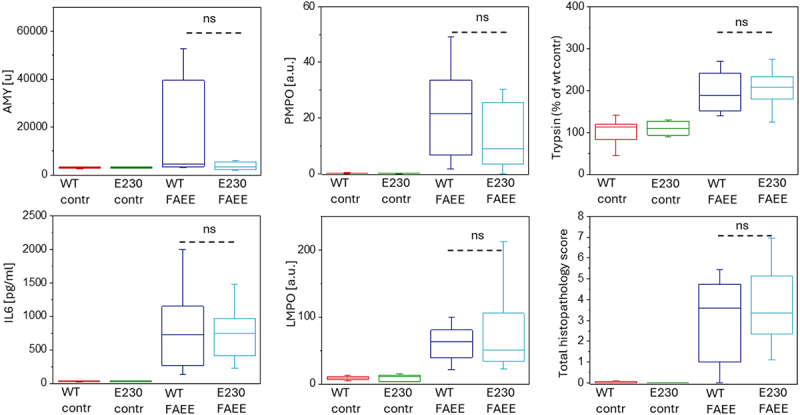
Severity of acute pancreatitis induced by palmitoleic acid and ethanol in wild-type and ATG16L1[E230] mice. The figure shows parameters characterizing the severity of acute pancreatitis in WDD and LNCA-deficient ATG16L1[E230] mice (abbreviated to E230 on the graph) and wild-type littermates (WT). Experimental acute pancreatitis was induced by two hourly intraperitoneal injections of 1.35 g/kg ethanol and 150 mg/kg palmitoleic acid (POA) this type of experimental pancreatitis is abbreviated as the FAEE model; 11 ATG16L1[E230] mice and 11 WT mice were utilized in these experiments. Animals were humanely sacrificed 24 h after the first injection. Control experiments involved intraperitoneal injections of vehicle solution (5 ATG16L1[E230] mice and 5 WT mice were used). Symbols above the bars illustrate the outcome of a Mann-Whitney test; symbol ns indicates that the difference was not statistically significant (p > 0.05). We did not observe significant difference in any of the measured parameters. Specific *p* values were the following: for serum AMY p = 0.09, for pancreatic MPO (PMPO) p = 0.19, for pancreatic trypsin p = 0.55, for IL6 p = 0.89, for lung MPO (LMPO) p = 0.99, for total histopathology score p = 0.44; *p* values for the components of the total histopathology score were the following: for edema p = 0.99 (not shown), for leukocyte infiltration p = 0.61 (not shown) and for necrosis p = 0.5 (not shown).

We next probed the putative role of WDD deletion in fatty acid (palmitoleic acid (POA)) and ethanol model (abbreviated as FAEE model [50]) of acute pancreatitis. We applied this model to WT and to WDD and LNCA-deficient ATG16L1[E230] mice; acute pancreatitis was induced in both types of mice. Notably, in contrast to the caerulein model, there were no resolvable differences in any of the parameters characterizing the severity of experimental acute pancreatitis between WT and ATG16L1[E230] mice (Figure 6) in the FAEE model. It is likely that the contribution of LNCA and WDD to acute pancreatitis cannot be revealed in this model because LNCA of the endocytic vacuoles is strongly inhibited by the fatty acid.

## Discussion

LNCA is a recently identified form of non-canonical autophagy characterized by very rapid LC3 conjugation to the single membrane of endocytic vacuoles, which develop in pancreatic acinar cells treated with the inducers of AP [33]. The results of the current study confirmed, that similarly to LAP and CASM [39,40,43,44,51], LNCA requires a functioning WDD of ATG16L1; genetic deletion of the WDD results in the loss of LNCA and increase in the severity of caerulein -induced AP, suggesting that LNCA plays a protective role in AP. Notably, this was completely opposite to the original hypothesis underlying this project. The discovery of the fast non-canonical autophagy, which is initiated simultaneously with rapid trypsinogen activation, impelled us to formulate and test a modified co-localization hypothesis. The original colocalization hypothesis (e.g [52–54]. reviewed in [55,56] is an important theoretical concept in pancreatology, stating that “co-localization of lysosomal hydrolases with digestive enzyme zymogens plays a critical role in permitting the intracellular activation of digestive enzymes” [56]. The rapid LC3 conjugation to endocytic vacuoles [33] and the presence of trypsin in the LC3-coated vacuoles [33], observed in the previous study, suggested the possibility that the trypsinogen activation is initiated in LNCA-mediated, non-canonical autolysosomes (derived from the endocytic vacuoles) and therefore we hypothesized that LNCA is required for trypsinogen activation. The results of the current study suggest the opposite effect of LNCA. The absence of the resolvable difference in the early peak value of the pancreatic trypsin content, observed in the single caerulein injection model, strongly suggests that LNCA is not required for trypsinogen activation (i.e. trypsin formation). On the contrary, the major reduction in the pancreatic trypsin recovery/decline rate in the LNCA-deficient mice and pancreatic acinar cells indicates that LNCA serves as an early protective mechanism necessary for rapid trypsin degradation. Our findings are therefore consistent with the conclusions of several previous studies, suggesting a protective role of autophagy in pancreatitis ([19,20,57,58] reviewed in [35]), with a caveat that the earliest (and probably the major) form of protection is likely mediated by LNCA.

While the fast trypsin degradation requires LNCA, the slower trypsin clearance occurs in LNCA-deficient mice (Figure 4A). In this animal model trypsin could be degraded by enzymes intrinsic to zymogen granules (e.g., CTSL [cathepsin L] [59]) and likely retained in the endocytic vacuoles [33] or undergo autodegradation (autolysis) [60]. These processes could be independent of LNCA and contribute to the slow decline of trypsin levels observed in WDD and LNCA-deficient ATG16L1[E230] mice. It is also conceivable that slower forms of autophagy (e.g [48]) play a significant role in slow trypsin clearance recorded at 4 and 6 h in LNCA-deficient mice. Another possible mechanism of this slow trypsin clearance from the pancreas (or pancreatic acinar cells) of LNCA-deficient mice could involve secretion of trypsin via exocytosis of endocytic vacuoles. This “secondary” exocytosis has been shown to occur at both apical and, notably, basolateral sites [24]. In this way trypsin can be removed from the pancreatic tissue/acinar cells and delivered to both pancreatic ducts and the interstitial fluid. Elucidation of the specific LNCA-dependent and independent mechanisms and their contribution to the fast and slow trypsin clearance are the subjects of further investigation.

It is conceivable that LNCA is already functioning as a protective mechanism at the physiological level of stimulation. Endocytic vacuoles and LC3-conjugated endocytic vacuoles were occasionally observed at the upper physiological levels of CCK (10 pM) (although both the numbers of the vacuoles and the percentage LC3-conjugated vacuoles increase significantly for the higher CCK concentrations) [24,33]. One can envisage that even in physiological conditions the cells occasionally generate excessive fusion of secretory granules during compound exocytosis peculiar for this cell type and that LNCA serves as quality control mechanism selectively triggered by the digestive enzymes retained and activated in endocytic vacuoles. Protective effects of LNCA in the moderate and mild forms of experimental AP are consistent with this notion. LNCA is a remarkably fast form of autophagy [33] and we observed a prominent effect of LNCA on the fast trypsin recovery rate after a single caerulein injection. However, our experiments also indicate that LNCA plays protective role at later time points of experimental AP. We observed a significant difference in trypsin level at 8 h time point of “standard” multi-injection models. One can envisage that a wave of pancreatic LNCA activation accompanies each caerulein injection in the multi-injection model of AP. It is also conceivable that at the later time points LNCA “collaborates” in exerting its protective effect with other slower forms of autophagy that have been shown to develop in pancreatic acinar cells (e.g [48]). Another putative explanation of the outcome from these experiments is related to the recently-identified WDD-dependent role of ATG16L1 in the regulation of cytokine responses [61]; this could be particularly relevant for the multi-injection caerulein models. The outcomes of rapid single injection cellular and animal models are less likely to be influenced by changes in cytokine responses. The relative contributions of these two WDD-dependent pathways (characterized by changes in the cytokine responses and by effects on rapid trypsin degradation) will be the subject of further investigations.

Fatty acids are amongst known precipitants and aggravators of acute pancreatitis (e.g [50,62]. reviewed in [63,64]). Our cellular-level experiments with POA, conducted in preparation for an additional POA-based FAEE model of acute pancreatitis, resulted in a surprising finding that LNCA is strongly suppressed by this fatty acid. This finding provides a putative explanation for the outcomes of our experiments with *in vitro* and *in vivo* models of FAEE pancreatitis in which no differences between WT and ATG16L1[E230] were resolved. Inhibition of a protective mechanism is likely to serve as a damaging/aggravating factor, suggesting a novel mechanism in pancreatic lipotoxicity.

The events preceding the LNCA activation include the formation of endocytic vacuoles. Pancreatic acinar cells utilize compound exocytosis, involving intergranular fusion [65]. Ca^2+^-releasing inducers of AP trigger aberrant excessive fusion and formation of giant structure composed from multiple fused secretory granules [23,24]. These structures are temporarily connected to the plasma membrane via the walls of the fusion pore and the pore mediates the secretion of zymogens. The fusion pore in the pancreatic acinar cells has a prolonged opening time [66]. It is, however, rather delicate; it restricts movement of molecules with molecular mass more than 70 kDa [24] and is likely to limit the flow of substances destined for exocytosis. After a few minutes of secretion, the pore closes and the postexocytic structure disconnects from the plasma membrane, forming an endocytic vacuole [23,33]. Importantly the endocytic vacuoles retain constituents of zymogen granules (e.g. AMY [33]), suggesting that it disconnects before secreting/releasing its complete content. Formation of trypsin in the endocytic vacuoles is the likely consequence of trypsinogen retention.

Currently the trigger for LNCA is unknown. An attractive hypothesis is that LNCA is specifically induced by the endocytic vacuoles that retain a large quantity of zymogens and are in danger of trypsinogen activation or by vacuoles that have actually activated trypsinogen (formed trypsin). This would provide the cell with an efficient mechanism to utilize the available LC3 and LNCA capacity to deal with the foci of trypsinogen activation. Furthermore, this could explain how the relatively small proportion of LC3-coated vacuoles (10–20% of all endocytic vacuoles observed at 30 min after CCK stimulation) can have such a significant effect on the trypsin dynamics. Another putative explanation is that all endocytic vacuoles undergo LNCA and what we observe is a snapshot of non-canonical autophagic flux (i.e. most of endocytic vacuoles undergo LNCA but only 10–20% are at the LC3-coated stage, the rest are either not yet LC3 coated or have already lost the LC3) in the progression to autolysosomes. In this scenario LNCA can process all endocytic vacuoles and contribute to trypsin degradation in all endocytic vacuoles. There is evidence of trypsin in LC3-coated endocytic vacuoles [33] and of endocytic vacuoles progression through the autophagic flux [23,33]. Relationships between the retention of zymogens, LNCA and LNCA-mediated non-canonical autophagic flux are important avenues for further investigation.

Our study demonstrates that even the earliest form of autophagy (LNCA) is not required for trypsinogen activation in the endocytic vacuole. This supports the notion that early trypsinogen activation does not involve input from other organelles and develops autonomously due to the activity of enzymes retained in the endocytic vacuole (and originally present in zymogen secretory granules). CTSB (cathepsin B) is a candidate for such an activating role [67,68]. Pancreatic acinar cells employ several mechanisms reducing/preventing intrapancreatic trypsinogen activation and dealing with its consequences (reviewed in [69]). Notably, trypsin can undergo autodegradation (autolysis) and can also be degraded by several proteases present in secretory granules or other cellular organelles (e.g [59,60,70]). The protective effect of LNCA could involve stabilizing the vacuolar membrane (i.e. preventing rapture) so that vacuolar trypsin can undergo degradation by proteases already present in the vacuole (including autolysis) or facilitating delivery of proteases capable of trypsin degradation.

Canonical autophagy has been reported as a protective mechanism in pancreatitis (e.g [15,19,58]. reviewed in [35]). The relative contribution of LNCA and canonical autophagy to the protection from trypsin mediated damage is therefore an interesting unresolved question. It is likely that during the initial period after the caerulein infusion LNCA, which is very fast, serves as the dominant protective mechanism. It is conceivable that later canonical autophagy plays a more significant role in both trypsin degradation (e.g. at 4–6 h after the caerulein injection) and, importantly, removal of organelles damaged during the initial harmful insult. Notably, LNCA and canonical autophagy share the same participating proteins (including ATG16L1 and LC3 see [33]); with limited content of these proteins the two forms of autophagy could be in competition. It is conceivable that in conditions in which the pancreas experiences a single damaging event (or infrequent damaging events) the same ATG proteins sequentially participate, initially in LNCA and later in canonical autophagy.

This report focuses on the description of the role of WDD of ATG16l1 in LNCA, trypsin degradation and the regulation of acute pancreatitis severity. The cellular and molecular mechanisms mediating the observed phenomena and their links with zymogen retention as well as their relationships with other protective mechanisms are the subject of future investigation in our laboratory.

## Materials and methods

### Materials

The following compounds were used: Boc-Gln-Ala-Arg-MCA trypsin substrate (Enzo Life Sciences, BML-P237-0005); CCK (cholecystokinin) fragment 26–33 (Sigma-Aldrich, C2175); CellEvent™ Caspase-3/7 (ThermoFisher Scientific, R37111); collagenase (Sigma-Aldrich, C9407); Dextran, Texas Red™, 3000 MW, Neutral (ThermoFisher Scientific, D3329); HEPES (Sigma-Aldrich, H3375); hexadecyltrimethylammonium bromide (Sigma-Aldrich, H9151); linoleic acid (Cayman Chemical, CAY90150); mini protease inhibitor cocktail (cOmplete^TM^) from Roche Diagnostics (Sigma-Aldrich 11836153001); mouse IL6 Quantikine ELISA Kit (R&D systems, M6000B); palmitoleic acid (Cayman Chemical, CAY10009871); propidium iodide (ThermoFisher Scientific, P3566); tetramethylbenzidine (Sigma-Aldrich 860336).

### Animals and procedures

All animal studies were ethically reviewed and conducted according to UK Animals (Scientific Procedures) Act 1986, approved by UK Home Office (PDC14C46E) and the University of Liverpool Animal Welfare Committee and Ethical Review Body (AWERB; AWC0145).

GFP-LC3#53 mice (referred to as GFP-LC3 mice) were originally developed by N. Mizushima and colleagues [71] and provided by the RIKEN BRC through the National Bio-Resource Project of the MEXT, Japan. RIKEN RRC reference number for these animals is RBRC00806. For the generation of GFP-LC3 mice founder animals (C57BL/6N Crj x BDF1) expressing GFP-LC3 were backcrossed to C57BL/6N Crj and maintained as heterozygotes for the gene of interest [71].

Animals were housed and bred in the Biomedical Services Unit at the University of Liverpool and had *ad libitum* access to food and water.

For one experiment involving the rapid caerulein model (see the Results section) we utilized overnight fasting. In this case mice had free access to water, however, food was withdrawn for 12 h before the beginning of the experiment.

ATG16L1[E230] mice [43,44] and corresponding WT littermates were available from Professor Ulrike Mayer and Professor Thomas Wileman (University of East Anglia, UK). For the generation of ATG16L1[E230] mice founder animals (C57BL/6N Crl x 129 Sv) expressing ATG16L1[E230] were backcrossed to C57BL/6J and maintained as heterozygotes for the genes of interest [43,44].

GFP-LC3#53 ATG16L1[E230] mice (referred to GFP-LC3 ATG16L1[E230] mice in text and GFP-LC3 E230 in figures) were generated in the Biomedical Services Unit at the University of Liverpool by crossing GFP-LC3#53 and ATG16L1[E230]. The generated homozygotes (for both GFP-LC3#53 and ATG16L1[E230]) were then utilized for further breeding. Genotyping was conducted by Transnetyx (Transnetyx, Inc. 8110 Cordova Rd. Suite 119, Cordova, TN 38,016, USA) using real time PCR. Fluorescence intensity was additionally used in our laboratory to verify GFP presence/content. GFP-LC3 ATG16L1[E230] mice had no obvious phenotypical changes in comparison with ATG16L1[E230] mice. Upon CCK stimulation, pancreatic acinar cells isolated from GFP-LC3 ATG16L1[E230] mice produced similar numbers of endocytic vacuoles to those from GFP-LC3 cells (see the Results section).

Animals were sacrificed by the Schedule 1 method of cervical dislocation, in accordance with the Animal (Scientific Procedures) Act 1986 (ASPA) and with approval by the University of Liverpool Animal Welfare Committee and Ethical Review Body (AWERB; AWC0145). Both female and male mice were used, at age 7–12 weeks.

### Visualizing endocytic vacuoles and LC3 conjugation to endocytic vacuoles

Only a negligible number of endocytic vacuoles form in unstimulated pancreatic acinar cells [33]. Endocytic vacuoles and LC3 conjugation to endocytic vacuoles were studied in cells stimulated by supramaximal concentrations (see the Results section) of cholecystokinin fragment 26–33 (CCK) at 34.5–35.0°C. This potent Ca^2+^-releasing agonist triggers secretion in pancreatic acinar cells [46,65,72]. Endocytic vacuoles were formed as a result of aberrant compound exocytosis of secretory granules followed by the membrane retrieval. To reveal endocytic vacuoles the cell-impermeable fluorescent indicator Dextran Texas Red 3000 MW was added to the extracellular solution at the time of CCK addition. LC3 conjugation to the endocytic vacuoles was revealed by GFP fluorescence in cells isolated from GFP-LC3 or GFP-LC3 ATG16L1[E230] mice.

Confocal fluorescence microscopes LSM 510 and LSM 710 (Zeiss, Oberkochen, Germany) were utilized in our experiments. We used objectives 63× with 1.4 numerical aperture (oil-immersion). Texas Red was excited with a 543 nm laser line and emission collected between 560 and 630 nm. GFP-LC3 was excited with a 488 nm laser line and emission collected between 500 and 530 nm.

A recent study by O. Mareninova and colleagues reported that GFP-LC3 expression slows down autophagic flux and increases the number of LC3 puncta [34]. This is an important observation, which suggested caution with using GFP-LC3 expression to study autophagy in pancreatic acinar cells. The observed increase of LC3-conjugated structures (approximately 2.5 times in comparison with WT mice) in this study was attributed to the change in the rate of resolving the autophagosomes via subsequent steps of the autophagic flux [34]. In our experiments we used GFP-LC3 mice and WDD-deficient GFP-LC3 ATG16L1[E230] mice to study GFP-LC3 conjugation to the endocytic vacuoles at a very early time point (30 minutes). Almost complete inhibition of GFP-LC3 conjugation to the endocytic vacuoles (i.e. inhibition of LNCA) in the acinar cells from WDD-deficient GFP-LC3 ATG16L1[E230] mice cannot be attributed to GFP-LC3 expression (both mouse types in our experiments express GFP-LC3) and strongly suggest a critical role of the WDD in LNCA. This finding is also consistent with the previously reported complete inhibition of LNCA by bafilomycin [33], which is a common property of LNCA and other forms of CASM (e.g [73]). While we utilized GFP-LC3 and GFP-LC3 ATG16L1[E230] mice to study the role of the WDD in LNCA, experiments with animal and cellular models of acute pancreatitis utilized WT and ATG16L1[E230] mice (i.e. animals that did not express GFP-LC3).

### Experimental AP models

AP was induced by seven intraperitoneal injections of caerulein at hourly intervals (see [74] for further information about this AP model). The effect of LAP-like non-canonical autophagy was evaluated using severe (50 μg/kg per injection), moderate (25 μg/kg per injection) and mild (10 μg/kg per injection) caerulein AP models. The numbers of WT mice and ATG16L1[E230] mice utilized in these experiments are described in the Results section. In control experiments (conducted for each AP model) mice received saline injections under the same conditions but without an AP inducer (5 mice were used in each control experiment). For this AP and its controls, mice were humanly sacrificed at 8 h after the first caerulein injection.

In this study we also investigated changes in pancreatic trypsin levels following a single intraperitoneal caerulein (50 μg/kg) injection. Mice were sacrificed before the first injection (corresponding to time 0) as well as one, two, four and six h after the injection. In the fatty acid (palmitoleic acid (POA)) and ethanol model (FAEE model [50]) of acute pancreatitis we utilized two hourly intraperitoneal injections of 1.35 g/kg ethanol and 150 mg/kg palmitoleic acid (POA) as described in [50]. Animals were humanely sacrificed 24 h after the first injection. Control experiments involved intraperitoneal injections of vehicle solution.

The numbers of WT mice and ATG16L1[E230] mice utilized in the animal models are described in the Results section.

### Enzyme activity and IL6 measurement

Pancreatic trypsin activity was measured using Boc-Gln-Ala-Arg-MCA substrate (380/440 nm excitation/emission; Enzo Life Sciences, BML-P237-0005) as described previously [75]. MPO activity was determined using tetramethylbenzidine substrate (Sigma-Aldrich 860336) as previously described [76]. Serum AMY and IL6 were determined kinetically using a Roche automated clinical chemistry analyzer (GMI, Leeds, UK) and ELISA kit (R&D Systems, M6000B), respectively.

### Histopathology

Pancreatic tissue was fixed in 4% paraformaldehyde in PBS (137 mM NaCl, 2.7 mM KCl, 10 mM Na_2_HPO_4_, 1.8 mM KH_2_PO_4_, pH 7.4), paraffin-embedded and H&E stained. Pancreatic histopathological scoring was performed on 10 random fields (magnification × 200) by two independent investigators following the blinding procedure, grading 0–4 was utilized for edema, inflammatory cell infiltration and acinar necrosis, respectively [77]. The overall pancreatic histopathology score was the sum of the individual scores.

### Isolation of pancreatic acinar cells

Pancreata were excised from sacrificed animals by dissection. Acinar cells were isolated by collagenase digestion (0.14–0.16 mg/mL). For confocal fluorescence imaging isolated cells were seeded on poly-L-Lysine-coated glass-bottom 35-mm dishes (MatTek Corporation, P35G-0-14-C) and kept in extracellular solution (140 mM NaCl, 4.7 mM KCl, 1.13 mM MgCl_2_, 10 mM 4-[2-hydroxyethyl]-1-piperazineethanesulfonic acid [HEPES]; 10 mM D-glucose, 1.2 mM CaCl_2_, pH 7.4). Specific compounds added to this extracellular solution were indicated in the description of individual experiments.

### Measurements of trypsin activity in isolated pancreatic acinar cells

The isolated pancreatic acinar cells were equally distributed between the prospective time groups (0, 1, 2, and 4 h) and placed into 15 ml tubes containing 10 ml of standard extracellular HEPES-based buffer (described in section “Isolation of pancreatic acinar cells”) at 37°C. Each group of cells was then stimulated with caerulein 50 µg/l for the specified time. The cells then were centrifuged at 78 *g* and resuspended in 15 ml of fresh HEPES-based buffer and the centrifugation-resuspension cycle was immediately repeated. Finally, the cells were centrifuged and resuspended in 1 ml of the ice-cold MOPS-based buffer as used in the pancreatic tissue trypsin activity assay [75]. The following steps of homogenization, centrifugation and the enzyme activity measurement itself were done exactly as in pancreatic tissue trypsin activity assay (see section Enzyme Activity and IL6 measurements and [75]).

### Cell death assays

Fluorescence of the probes characterizing apoptosis or necrosis was measured on a POLARstar Omega Plate Reader (BMG Labtech, Germany) at 37°C using 96 well flat bottom plates as previously described [78]. Necrosis of the cells was measured using the fluorescent dye propidium iodide (10 µg/ml final concentration). Excitation/emission filters of 540/620 nm were used in these experiments. Notably, propidium iodide determines total cell death involving permeabilization of the plasma membrane. Defining the specific type of necrosis was not a primary objective of our study. In our manuscript we have used term “necrosis” to refer to all possible necrotic processes resulting in plasma membrane permeabilization. Apoptosis of the acinar cells was measured using CellEvent™ Caspase-3/7 at 1:100 dilution. Fluorescence developed as the result of caspase activation was measured using excitation 480 nm and emission 520 nm. Apoptosis and necrosis measurements were run in triplicates. The end-point readings of necrosis and apoptosis were done at 14 h. All fluorescence measurements for necrosis and apoptosis of the cells from ATG16L1[E230] mice were normalized to the corresponding measurements conducted on the cells from WT littermates.

### Statistical analysis

The data were tested for normality using a Shapiro-Wilk test. If a normality hypothesis could not be rejected, further comparisons were made using a two-tailed Student’s t-test. Mann-Whitney test was used to compare two groups of independent observations for the data not following a normal distribution. Statistical significance was set at *p* < 0.05. Statistically significant differences were indicated by * symbol on the graphs.

## Supplementary Material

Suppl LNCA Text Fig 7Aug vFb R4.docx

## Data Availability

The data that support the findings of this study are available from the first/corresponding author (MC) upon reasonable request.
