# Supplementary material for: Deletion of the WD40 domain of ATG16L1 exacerbates acute pancreatitis, abolishes LAP-like non-canonical autophagy and slows trypsin degradation
Source: Autophagy. 2024 Aug 31;21(1):210–22. doi: 10.1080/15548627.2024.2392478 (PMC11702947; doi:10.1080/15548627.2024.2392478)
Supplement: Suppl LNCA Text Fig 7Aug vFb R4.docx [file KAUP_A_2392478_SM0784.docx]

**SUPPLEMENTARY FIGURES**

**
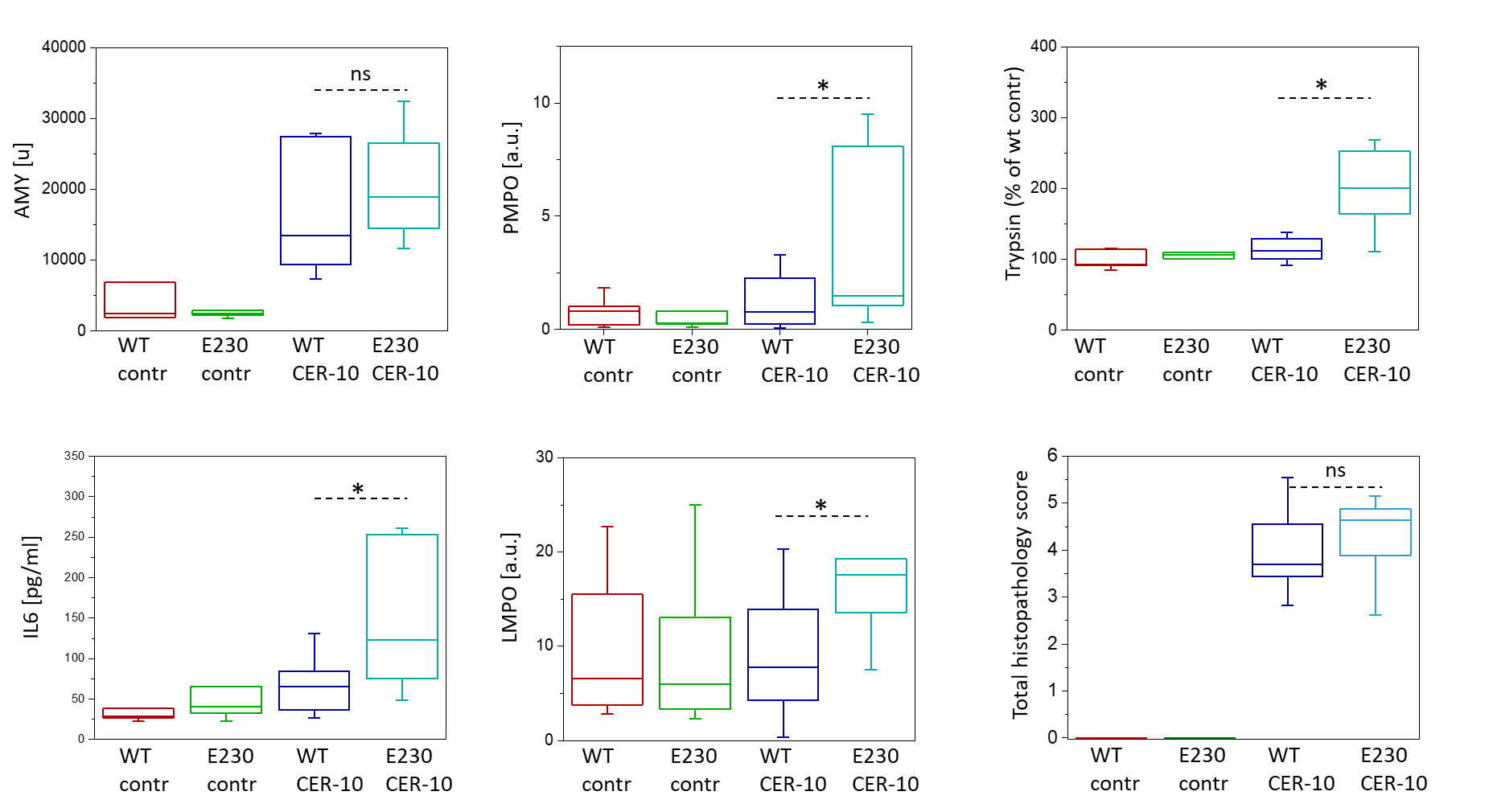
**

**Figure S1.** Severity of acute pancreatitis in wild-type and ATG16L1[E230] mice. Mild caerulein model (10 µg/kg). The figure shows parameters characterizing severity of acute pancreatitis (AP) in mice with deficient non-canonical autophagy (ATG16L1[E230] mice, abbreviated to E230 on the graph) and wild-type littermates (WT). Experimental AP was induced by 7 hourly intraperitoneal injections of caerulein (10 µg/kg); 11 ATG16L1[E230] mice and 11 WT mice were utilized in these experiments. Animals were humanely sacrificed 8 h after the first injection. Control experiments involved intraperitoneal injections of vehicle solution without caerulein (5 ATG16L1[E230] mice and 5 WT mice were used). Symbols above the bars illustrate the outcome of a Mann-Whitney test; symbol * indicates statistical significance (p<0.05), ns indicates that the difference was not statistically significant. Specific p values for experiments involving 10 µg/kg caerulein injections were the following: serum AMY p = 0.08, pancreatic MPO (PMPO) p = 0.026, pancreatic trypsin p = 0.005, IL6 p = 0.015, lung MPO (LMPO) p = 0.009, total histopathology score p = 0.44 (information about the components of the histopathology score is summarized on Figure S2).


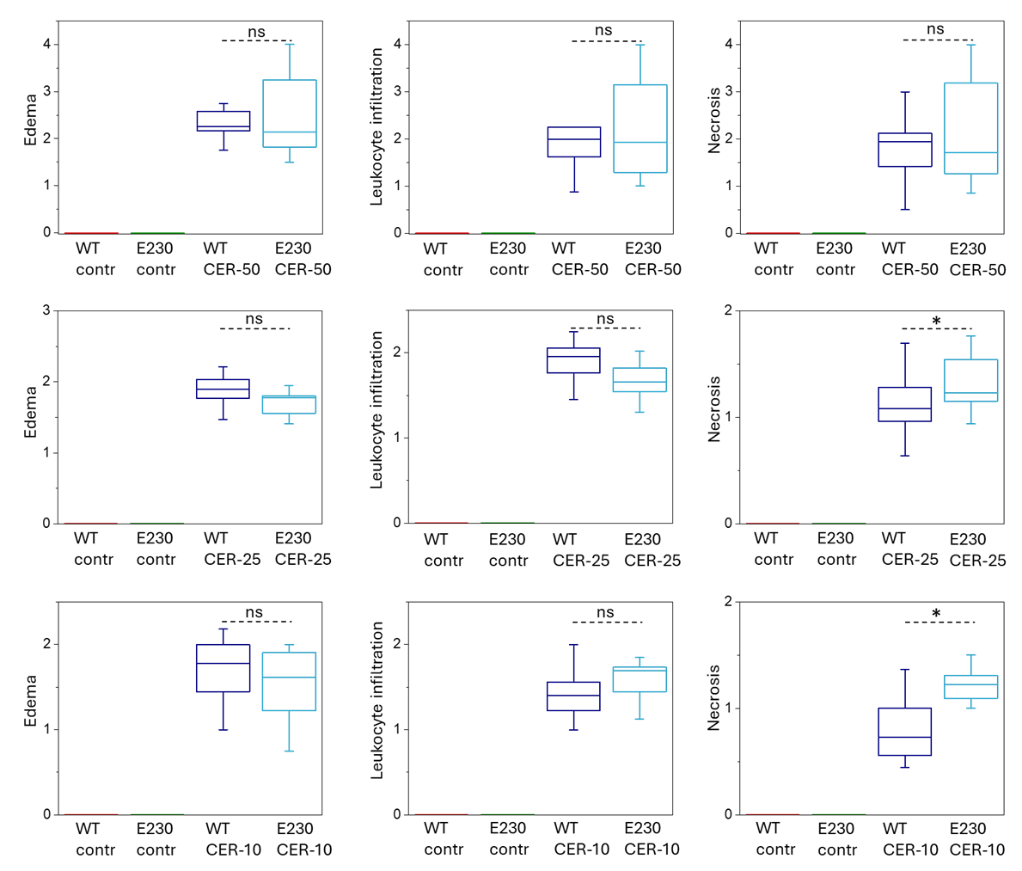


**A**

**B**


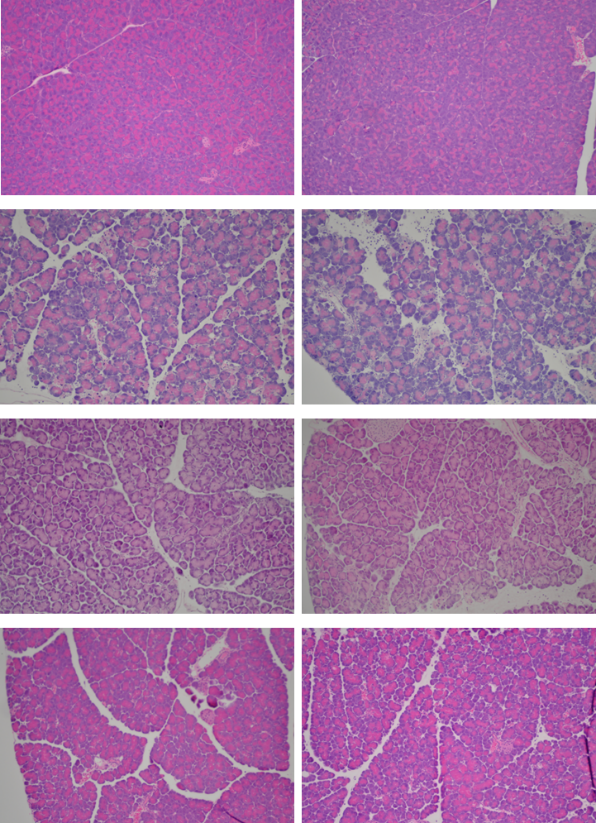


**WT control**

**E230 control**

**E230 Cer-50**

**WT Cer-50**

**E230 Cer-25**

**WT Cer-25**

**E230 Cer-10**

**WT Cer-10**

**Figure S2.** Individual histopathology scores and representative H&E-stained tissue sections for caerulein models of acute pancreatitis in wild-type and ATG16L1[E230] mice. Experimental conditions are described in the Methods section of the main manuscript, figure legend for Figure 1 of the main manuscript and figure legend for Figure S1. (**A**) Histopathology scores. Individual p values for the high caerulein model (50 µg/kg) were the following: edema p = 0.62, leukocyte infiltration p = 0.6 and necrosis p = 0.46. Individual p values for the moderate caerulein model (25 µg/kg) were the following: edema p = 0.1, leukocyte infiltration p = 0.14 and necrosis p = 0.044. Individual p values for the mild caerulein model (10 µg/kg) were the following: edema p = 0.26, leukocyte infiltration p = 0.3 and necrosis p = 0.002. (**B**) Representative H&E (hematoxylin and eosin) stained pancreatic tissue sections from WT and E230 mice subjected to intraperitoneal saline injections (control) or caerulein injections (in doses specified on individual slides). Scale bar: 200 µm (see the “WT control” panel).


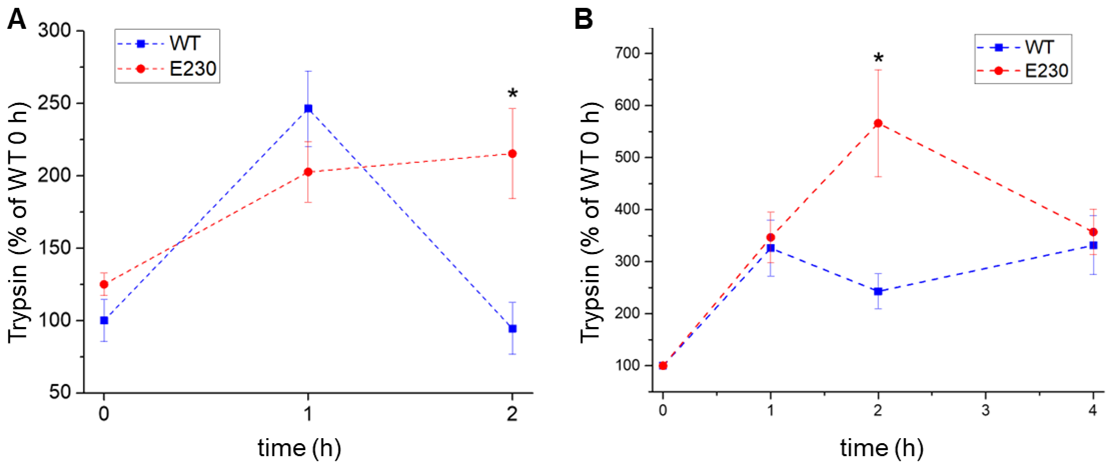


**Figure S3**. Deletion of WDD of Atg16L1 slows trypsin degradation in pancreata of fasted mice and in isolated pancreatic acinar cells. (**A**) Rapid changes in the pancreatic trypsin levels in pancreata of wild-type (WT) and ATG16L1[E230] (abbreviated to E230 on the graph) mice were induced by one intraperitoneal caerulein injection (50 µg/kg). Experiments were conducted on mice fasted for 12 h. Each time point on the graph shows the results of measurements (± standard error) normalized to the mean values from WT at 0 h for each of experiments. Symbol * indicates statistical significance (p < 0.05). Note the substantial and statistically significant decline in the trypsin levels between 1 h and 2 h in WT mice (p = 0.001), and the absence of such decline in the LAP-deficient E230 mice (p = 0.74). Trypsin levels at the 1 h time point were not significantly different between WT and E230 mice (p = 0.32) but became significantly different at the 2-h time point (p = 0.02). (**B**) Changes in the trypsin levels measured in pancreatic acinar cells isolated from pancreata of wild type (WT) and ATG16L1[E230] (abbreviated to E230 on the graph) mice. Cells were stimulated by caerulein (50 µg/l). Trypsin levels were measured in unstimulated cells (i.e. before the caerulein application, 0 h) as well as at 1 h, 2 h and 4 h after the caerulein application. Each time point on the graph shows the results of measurements (mean ± standard error). All results were normalized to the values obtained at 0 h for each of experiments. Symbol * indicates statistical significance (p < 0.05). The number of pancreata (and cell preparations) for 0 h were 12 WT and 7 E230, for 1 h were 12 WT and 7 E230, for 2 h were 12 WT and 7 E230, for 4 h were 7 WT and 5 E230. Trypsin levels at the 1 h time points were not significantly different between WT and E230 mice (p = 0.47). There was a statistically significant decrease in the trypsin levels between 1 h and 2 h in the acinar cells from WT mice (p = 0.03, blue trace). There was no statistically significant difference (p = 0.06) between 1 h and 2 h in the acinar cells from E230 mice (red trace). Notably, significant difference in the trypsin levels between the acinar cells from WT and E230 mice developed at 2 h (p = 0.02, indicated by symbol *) and disappeared at 4 h (p = 0.73) after the single injection.


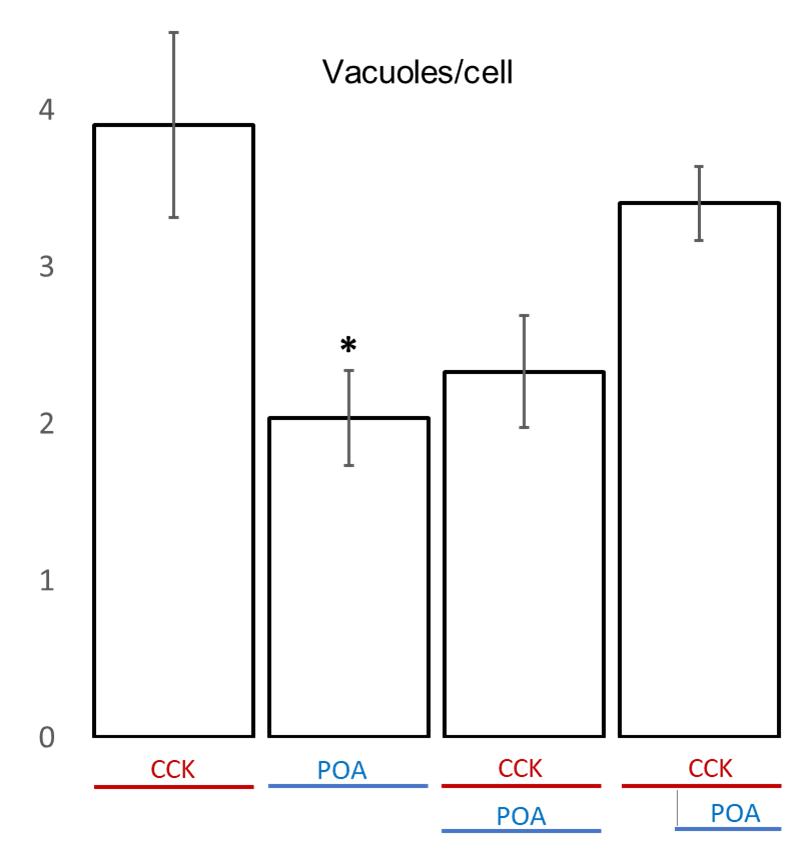


**Figure S4.** Effect of palmitoleic acid on the numbers of endocytic vacuoles in isolated pancreatic acinar cells. The figure shows the numbers of endocytic vacuoles per cell induced by the same treatments/compounds as in the Figure 5A of the main paper. The number of vacuoles produced by 200 µM POA was different from the number produced by 10 nM CCK (p = 0.025, calculated using Dunnett’s test for the experiments shown on this figure), while the number produced by 10 nM CCK and the delayed application of 200 µM POA was not significantly different from that produced by 10 nM CCK alone (p = 0.65). There was also no resolvable difference between the number of vacuoles produced by 10 nM CCK alone and the combination of 10 nM CCK with 200 µM POA applied simultaneously (p = 0.059).


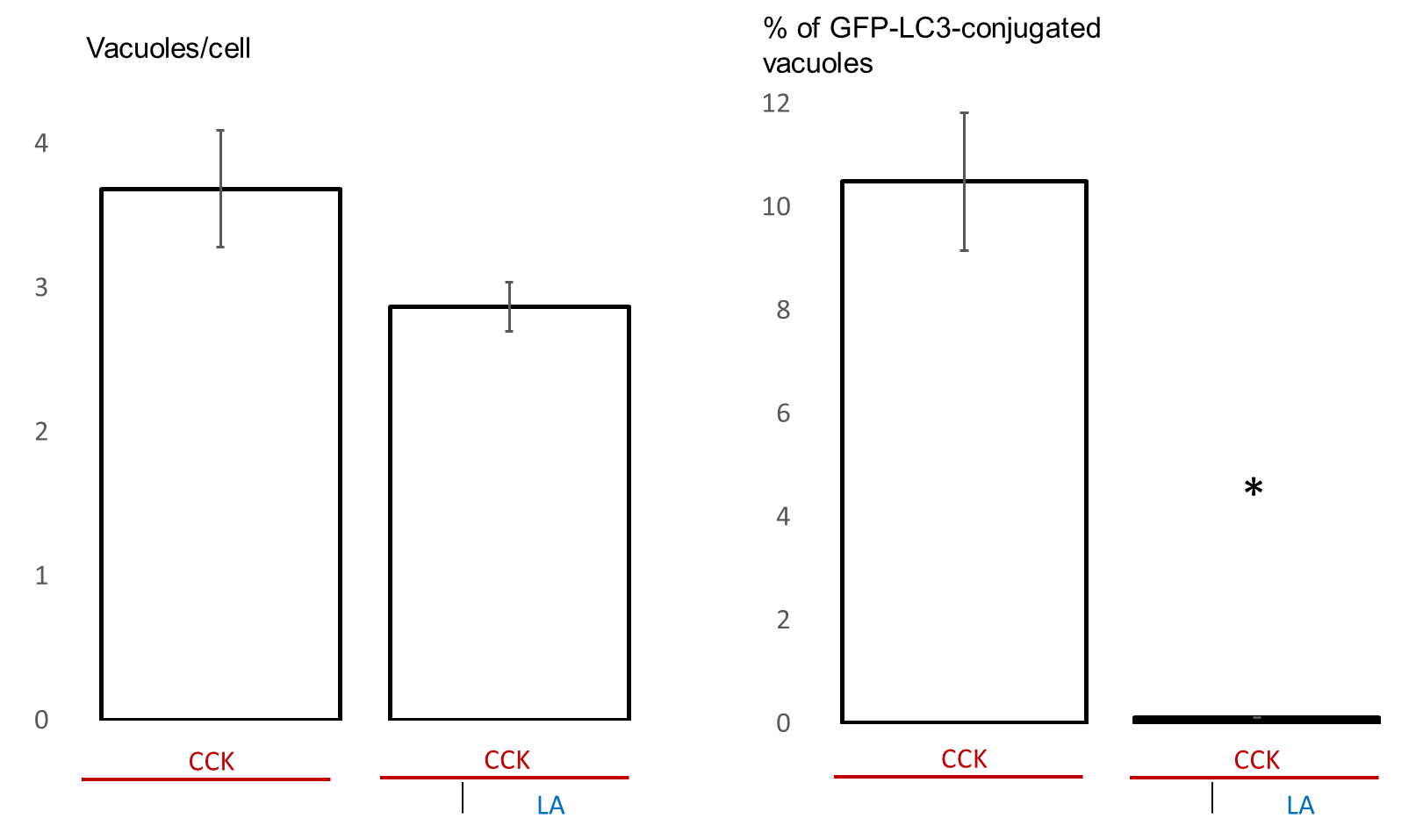


**Figure S5.** Effect of linoleic acid on the numbers of endocytic vacuoles and LAP-like non-canonical autophagy in isolated pancreatic acinar cells. The experiment involved application of 10 nM CCK followed by the application of LA 200 µM after a delay of 10 min (in the continuous presence of 10 nM CCK, LA was present for 20 min). Cells were isolated from GFP-LC3 transgenic mice a nd incubated in physiological HEPES-buffered solution containing Texas Red dextran (TRD) at 35^o^C. In all experiments shown in this figure the extracellular solution contained 1% ethanol (utilized as a vehicle for LA). In separate experiments (mentioned in the main text) we found that this ethanol concentration had no effect on the number of vacuoles (p = 0.97, not shown) and the proportion of GFP-LC3-conjugated vacuoles (p = 0.92, not shown) produced by 10 nM of CCK. Symbol * indicates statistically significant difference from the values obtained in experiments with 10nM CCK (without LA). Application of 10 nM CCK for 30 min resulted in the generation of endocytic vacuoles and GFP-LC3 conjugation to the endocytic vacuoles (665 vacuoles from 177 cells were analyzed, 9 mice were used for cell isolation in these experiments). Application of LA following 10-min incubation in CCK (112 vacuoles from 38 cells were analyzed, 4 mice were used in these experiments) had no resolvable effect on the vacuole number (p = 0.09) and drastic inhibition (right panel) of the GFP-LC3 conjugation to the endocytic vacuoles (p = 0.00003).


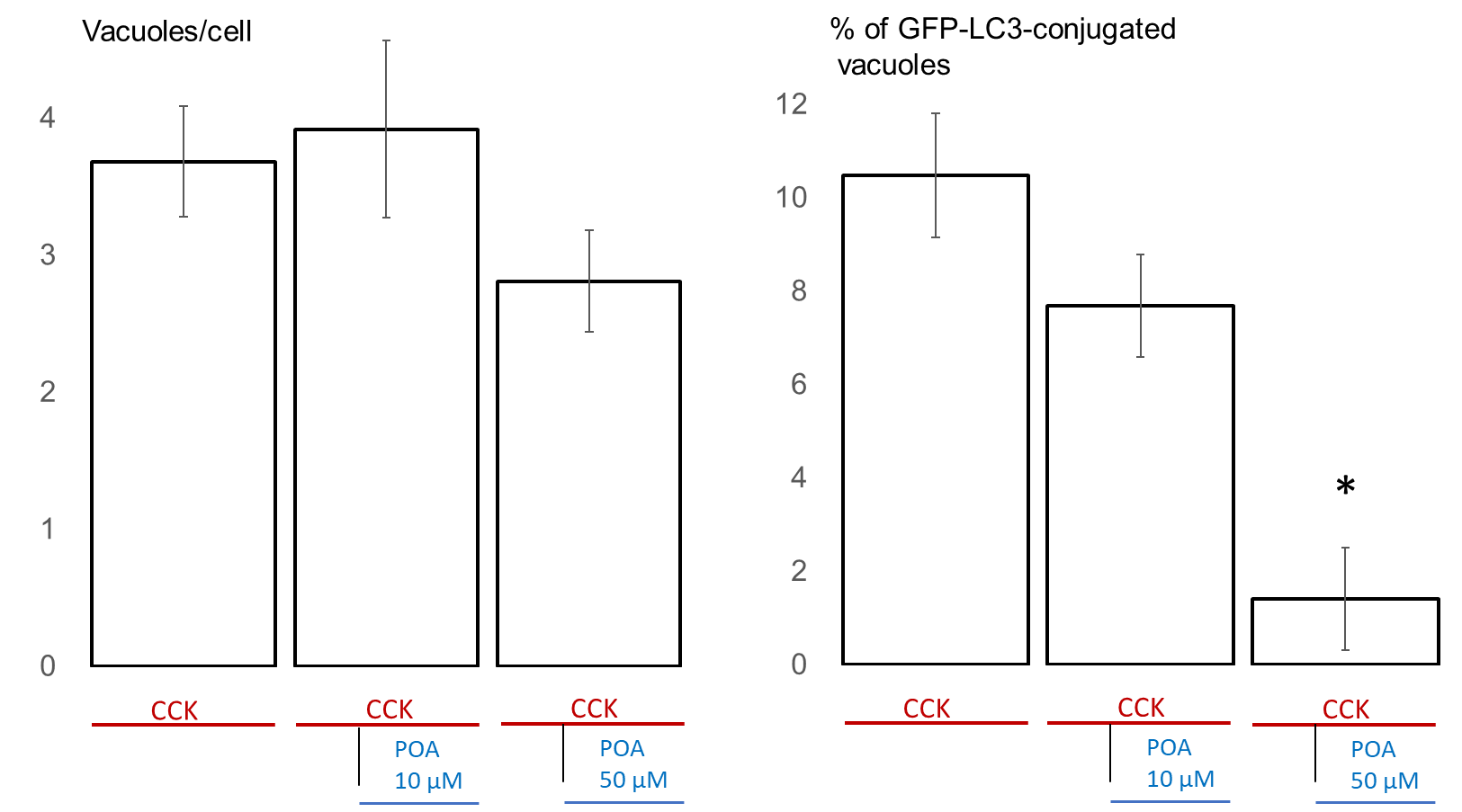


**Figure S6.** Effect of different concentrations of palmitoleic acid on the numbers of endocytic vacuoles and LAP-like non-canonical autophagy in isolated pancreatic acinar cells.

The experiment involved application of 10 nM CCK followed by the application of POA at concentrations 10 µM and 50 µM after a delay of 10 min (in the continuous presence of 10 nM CCK, POA was present for 20 min). Cells were isolated from GFP-LC3 transgenic mice and incubated in physiological HEPES-buffered solution containing Texas Red dextran (TRD) at 35^o^C. Symbol * indicates statistically significant difference from the values obtained in experiments with 10nM CCK (without POA). Application of 10 nM CCK for 30 min resulted in the generation of endocytic vacuoles and GFP-LC3 conjugation to the endocytic vacuoles (665 vacuoles, from 177 cells were analyzed, 9 mice were used for cell isolation in these experiments). Application of 10 µM POA following 10-min incubation in CCK (389 vacuoles, from 96 cells were analyzed, 6 mice were used in these experiments) had no resolvable effect on the vacuole number (left panel, p = 0.77); there was also no resolvable effect of 10 µM POA on the GFP-LC3 conjugation to the endocytic vacuoles (right panel, p = 0.12). Application of 50 µM POA following 10-min incubation in CCK (309 vacuoles from 113 cells were analyzed, 6 mice were used in these experiments) had no resolvable effect on the endocytic vacuole number (left panel, p = 0.14); 50 µM POA strongly suppressed GFP-LC3 conjugation to endocytic vacuoles (right panel, p = 0.0001). The results of experiments with 200 µM POA are shown on the Figure 5 of the main manuscript.
